# Supplementary material for: Inhibition of LNC EBLN3P Enhances Radiation-Induced Mitochondrial Damage in Lung Cancer Cells by Targeting the Keap1/Nrf2/HO-1 Axis
Source: Biology (Basel). 2023 Sep 4;12(9):1208. doi: 10.3390/biology12091208 (PMC10525126; doi:10.3390/biology12091208)

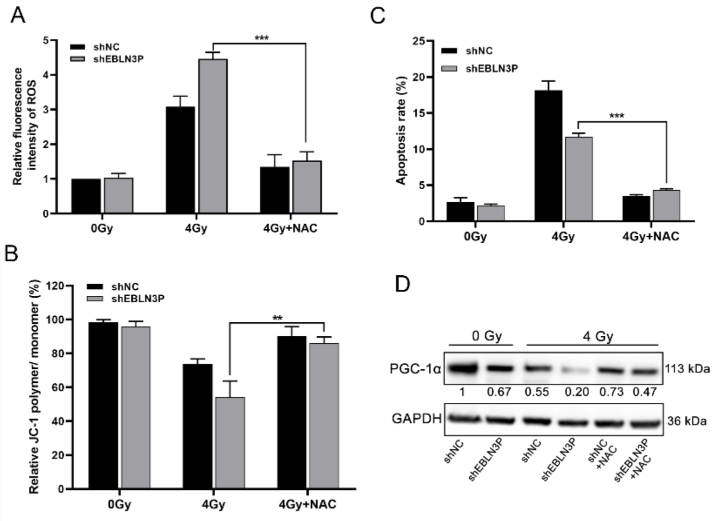

Figure S1. Evaluation of intracellular ROS levels, apoptosis, MMP and PGC-1α expression in H1299-shNC/shEBLN3P cells treated with NAC after exposure to 4 Gy of X-ray irradiation. (A) Intracellular ROS levels were evaluated in H1299 cells at 1 h after exposure to 4 Gy of X-ray irradiation. (B) Apoptotic cells were analyzed by flow cytometry. (C) Quantification of JC-1 staining for mitochondrial membrane potential (MMP). (D) Western blot analyses were conducted to determine the levels of PGC-1α. Relative densitometry values for the representative blots are given below each band. Data are represented as means ± SD (three biological replicates; $**p$ <0.01, $***p < 0.001$).

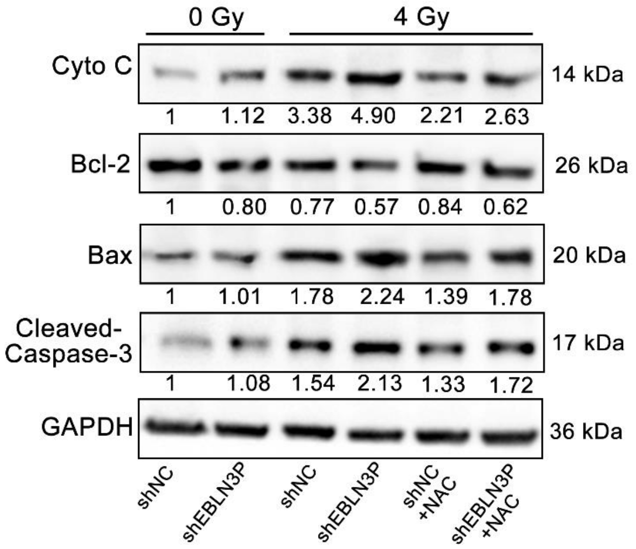

Figure S2. Cytochrome C (Cyto C), Bcl-2, Bax and cleaved Caspase-3 levels were evaluated in H1299 cells 24 h after irradiation by Western blot. Relative densitometry values for the representative blots are given below each band.

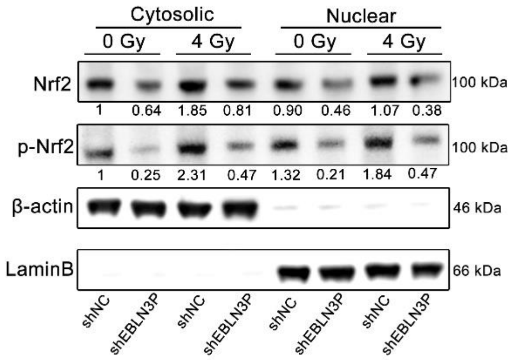

Figure S3. Nrf2 and its phosphorylation levels in cytoplasm and nucleus were assessed by Western blot analysis. Relative densitometry values for the representative blots are given below each band.

Figure S4. Original Western blot images for the Western blot experiments in Figures 2~5:

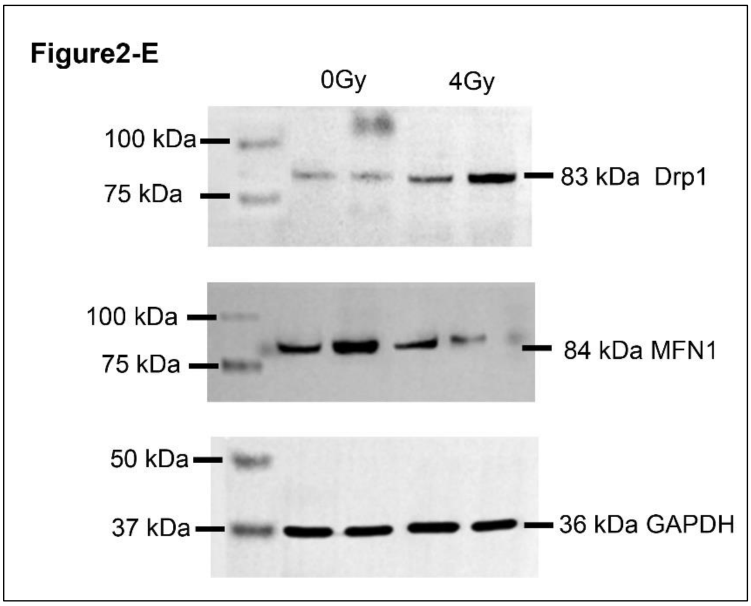

Figure3-D

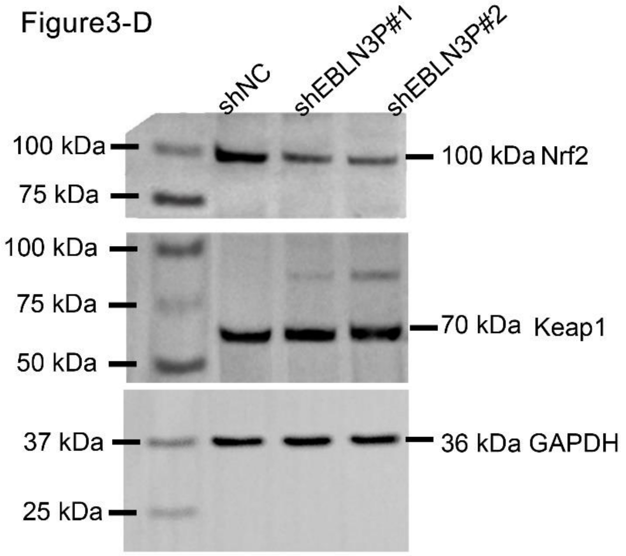

Figure4-A

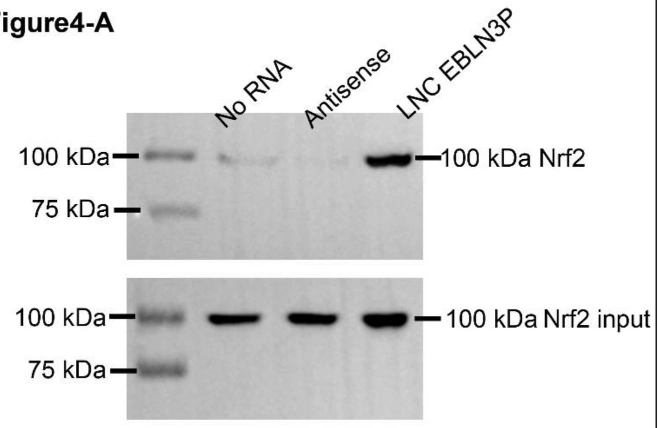

Figure4-C

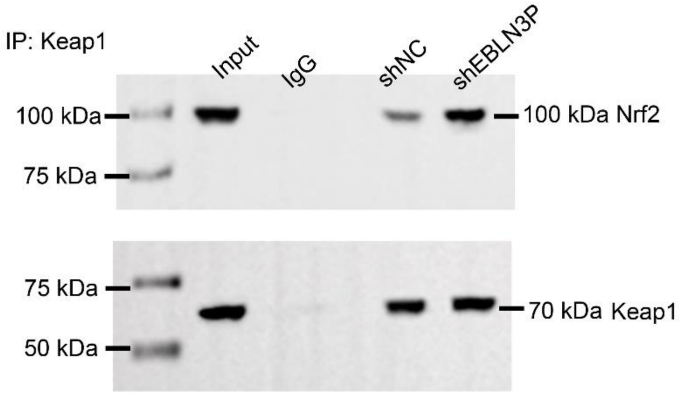

Figure4-D

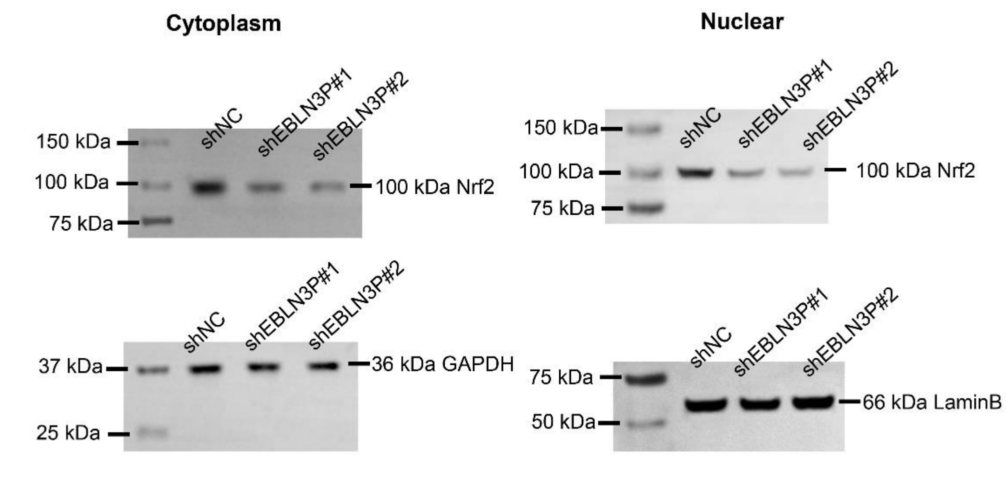

Figure5-C

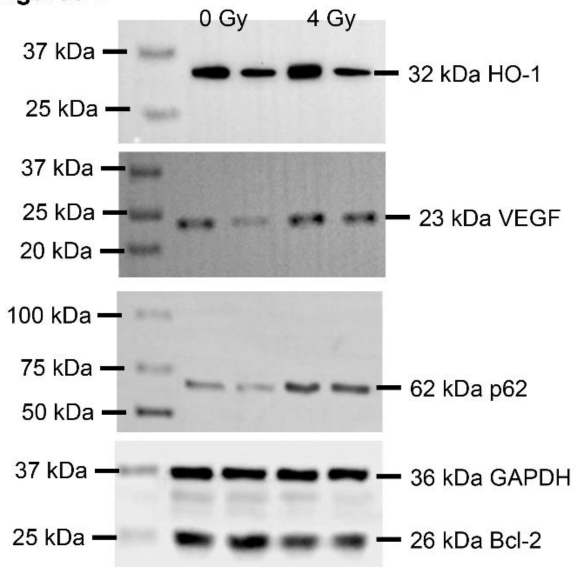

Figure5-D

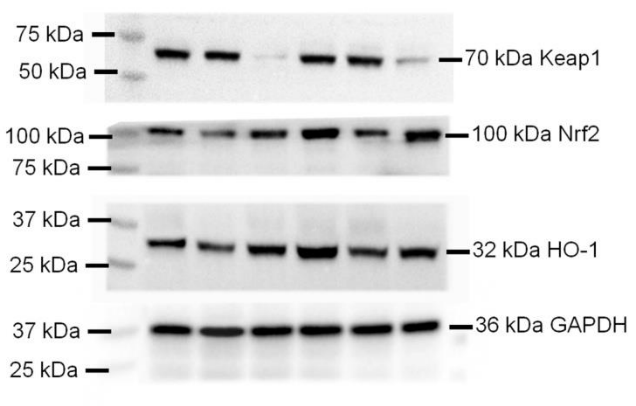

Supplement: Supplementary file 1 [file biology-12-01208-s001.zip › biology-12-01208-s001.pdf]
